# Low ETV1 mRNA expression is associated with recurrence in gastrointestinal stromal tumors

**DOI:** 10.1038/s41598-020-71719-y

**Published:** 2020-09-08

**Authors:** Keiichi Sakamaki, Kohei Funasaka, Ryoji Miyahara, Kazuhiro Furukawa, Takeshi Yamamura, Eizaburo Ohno, Masanao Nakamura, Hiroki Kawashima, Yoshiki Hirooka, Mitsuhiro Fujishiro, Hidemi Goto

**Affiliations:** 1 Department of Gastroenterology and Hepatology, Nagoya University Graduate School of Medicine, Nagoya, 466-8550 Japan; 2 Department of Gastroenterology, Fujita Health University School of Medicine, 1-98 Kutsukake-cho, Toyoake, Aichi 470-1192 Japan

**Keywords:** Tumour biomarkers, Prognostic markers, Sarcoma

## Abstract

Although the majority of gastrointestinal stromal tumors (GISTs) possess KIT mutations that induce constitutive signal transduction, the clinical outcomes are variable. The ETS translocation variant 1 (ETV1) gene encodes a transcription factor that is reported to cooperate with KIT in GISTs. However, the clinical role of ETV1 is largely unknown. The aim of this study was to examine ETV1 expression and its associations with clinical features in GISTs. We conducted a cohort study involving 64 patients with GISTs who underwent surgical resection between October 2008 and February 2015. ETV1 mRNA expression was compared with that in non-GISTs and was analyzed among risk classifications or clinical outcomes. The GIST samples exhibited significantly higher ETV1 mRNA expression than the non-GIST samples (*P* < 0.0001). Sixty-four GISTs were stratified into high or low ETV1 mRNA expression groups based on the median relative abundance of ETV1 mRNA. The multivariate analysis showed that low ETV1 expression, as well as tumor size and mitotic index, was an independent factor of recurrence (hazard ratio: 8.1). Patients with high ETV1 expression achieved significantly longer recurrence-free survival (RFS) times than those with low ETV1 expression (*P* = 0.025). Our study revealed that low ETV1 expression is an independent factor of recurrence after surgery in patients with GISTs, and thus, low ETV1 expression might be a marker of more aggressive malignant GISTs.

## Introduction

Gastrointestinal stromal tumors (GISTs) are the most common mesenchymal tumors of the gastrointestinal tract. Most of them specifically express the c-kit protein and carry mutations in the KIT or PDGFRA gene^1–3^. Gene mutations found in GISTs affect the constitutive activation of the c-kit receptor, which is closely related to tumorigenesis, and are classified as “gain-of-function” mutations^1,4–7^*.* In 2001, it was reported that the tyrosine kinase inhibitor (TKI) imatinib markedly suppressed the progression of unresectable GISTs by inhibiting the constitutive c-kit signaling pathway^8,9^. Therefore, KIT is considered a key molecule in the oncogenesis of GISTs.

In general, GISTs exhibit variable biological behaviors in the clinical setting. Some GISTs grow rapidly or produce liver metastases, which can lead to death^10^. On the other hand, some GISTs are found incidentally without any clinical symptoms, even though they have the same KIT mutations^11,12^. The relationships between clinical aggressiveness and gene mutations in GISTs remain unclear. Currently, gene mutations are recognized as an initiating factor of transformation to malignancy, but other factors are also required^13,14^. Thus, the discovery of new biomarkers that could be used to predict the malignancy and clinical course of GISTs is desired.

In 2010, it was reported that the transcription factor ETS translocation variant 1 (ETV1) is universally expressed in GISTs and contributes to tumorigenesis by cooperating with KIT^15^. However, other studies concluded that ETV1 is not expressed in all GISTs^16,17^. Moreover, there was no significant difference in the relapse-free survival rate between patients with GISTs that did and did not express ETV1^17^. Thus, the relationships between ETV1 expression and prognosis in patients with GISTs remain unclear. Thus, we conducted this study to evaluate ETV1 mRNA expression and to clarify the relationships between ETV1 mRNA expression and risk classifications or clinical significance in GISTs.

## Materials and methods

### Patient cohort

From October 2008 to February 2015, we registered 64 consecutive patients who underwent surgery for GISTs at Nagoya University Hospital. GISTs were categorized as very low-, low-, intermediate-, and high-risk tumors according to the modified National Institutes of Health (NIH) consensus criteria^18^. In March 2020, medical records were surveyed to determine whether the patients had suffered recurrence and whether they had survived (median interval: 60 months; range 2–96 months). Protocols for the collection of tumor samples and clinical information were approved by the Institutional Review Board at Nagoya University Hospital. Written informed consent was obtained from all patients before surgery. All methods were performed in accordance with the relevant guidelines and regulations.

### RNA extraction and cDNA reverse transcription

In each case, a tissue with a side of 5 mm was cored out from the resected fresh tumor in the operating room and immediately immersed in RNAlater stabilization solution (Invitrogen, Carlsbad, CA). The sample was stored at − 80 °C until just before the total RNA extraction procedure using TRIzol reagent (Invitrogen, Carlsbad, CA), chloroform, and isopropanol according to the manufacturer’s instructions. For cDNA synthesis, 300 ng of total RNA was reverse transcribed using PrimeScript Reverse Transcriptase (Takara Bio, Inc., Shiga, Japan) according to the manufacturer’s protocol.

### Mutation analysis

Full-length KIT cDNA was amplified with over 40 cycles of polymerase chain reaction (PCR) using appropriate primers (Table S1). The amplified full-length KIT cDNA was used as a template, and exons 8, 9, 11, 13, and 17 were analyzed using the direct sequencing method on an ABI 310 system (Applied Biosystems, Foster City, CA). In patients who were negative for KIT mutations, exons 12, 14, and 18 of the platelet-derived growth factor receptor A (PDGFRA) gene were further analyzed after PCR amplification of the full-length PDGFRA cDNA. GISTs that did not possess KIT or PDGFRA mutations were defined as wild-type GISTs. If no mutation was detected in either KIT or PDGFRA using cDNA, we also performed mutation analysis by conventional analysis using DNA^6,13,19^ and confirmed whether wild-type GISTs were present.

### Quantitative real-time PCR

cDNA was synthesized starting from 100 ng/µl of total RNA. The expression level of each gene was analyzed with the StepOne real-time PCR system (Applied Biosystems, Foster City, CA). The following primers were used: ETV1 (Hs 00951941_m 1) and GAPDH (Hs 02758991_g 1). GAPDH expression served as an internal control. The sample that showed the lowest ETV1 mRNA expression was set as a reference. The 2^-ΔΔ*CT*^ method was used to calculate relative gene expression levels during direct comparisons between each of the samples. To compare ETV1 mRNA expression with GIST samples, 13 non-GIST samples that were preoperatively suspected as a GIST (one pancreatic ductal cancer, one metastatic lesion of cecal cancer, one gastric cancer, five schwannomas and five leiomyomas) were also analyzed for ETV1 mRNA expression by the same method. All experiments were performed in duplicate.

### Western blot analysis

Protein lysates from frozen tumor tissues were prepared in RIPA buffer (Cell Signaling, Danvers, MA). Twenty micrograms of protein in sample buffer was resolved by 8% SDS-PAGE, blotted onto a PVDF membrane, and processed for Western blotting. As primary antibodies, mouse monoclonal ETV1 (clone 2A8, 1:1,000; Abnova, Taiwan) and rabbit monoclonal βactin (4970S, 1:1,000, Cell Signaling Technology, Danvers, MA) were utilized.

### Statistical analysis

In the statistical analyses, the comparison of two groups was performed using the Mann–Whitney U test. Univariate and multivariate analyses regarding recurrence or survival after surgery were performed using the Cox proportional hazards model. The Kaplan–Meier method was then used to calculate estimates of the proportions of patients who were alive and free of recurrence. Overall survival (OS) and recurrence-free survival (RFS) rates were compared between the high and low ETV1 mRNA expression groups using the log-rank test. Patients who had not experienced either event at the last follow-up were censored. Statistical significance was defined as *P* < 0.05. All statistical analyses were performed using the Statistical Package for the Social Sciences (SPSS) version 24 (IBM, Armonk, NY, USA).

### Ethical approval

This research involved human participants. Protocols for the collection of tumor samples and clinical information were approved by the Institutional Review Board at Nagoya University Hospital. Written informed consent was obtained from all patients before surgery.

## Results

### Patient characteristics

The clinical features of the 64 GIST patients are shown in Table 1. Among the 64 patients, 11 suffered from postoperative recurrence. Two of the six patients who underwent adjuvant therapy experience recurrence. We defined a GIST that recurred after surgery as an aggressive malignant GIST. All 11 patients who experienced recurrence were treated with TKIs such as imatinib, sunitinib, and regorafenib. However, five patients died of GISTs. The characteristics of these patients are described in Table S2.

**Table 1:** Characteristics of Patients with GISTs.

	All (n = 64)	Low ETV1 (n = 32)	High ETV1 (n = 32)
**Sex**			
Male	33	17	16
Female	31	15	16
Age, years, mean (SD)	64.0 ± 11.1	61.1 ± 11.8	66.8 ± 9.7
Tumor size (mm), median, range	30 (15–300)	36.5 (15–300)	26.5 (15–80)
**Tumor location**			
Stomach	61	30	31
Small intestine	3	2	1
**Mitotic count/5 mm**^**2**^			
< 5	50	21	29
5–10	3	3	0
> 10	11	8	3
**Risk classification**^**a**^			
Very low	14	5	9
Low	33	15	18
Intermediate	4	2	2
High	13	10	3
Adjuvant TKI therapy	6	5	1
**Postoperative recurrence**			
+	11	9	2
−	53	23	30
**Dead of GISTs**			
+	5	4	1
−	59	28	31

^a^Risk classification according to the modified NIH consensus criteria.

### Gene mutations in GISTs

The results of the mutation analysis are shown in Table 2. Fifty-eight GISTs (91%) carried KIT mutations, whereas two GISTs carried PDGFRA mutations. Four GISTs had neither KIT nor PDGFRA mutations and were confirmed by additional DNA analysis as wild-type GISTs. Regarding the mutation status of the 11 aggressive malignant GISTs, KIT exon 11 mutations were detected in 9 patients (deletions were detected in 6 patients, point mutations were detected in 2 patients, and one insertion was detected). A KIT exon 9 insertion was detected in one of these GISTs. The remaining aggressive malignant GISTs had wild-type KIT.

**Table 2:** The c-KIT and PDGFRA mutations in GISTs.

Type of mutation	No. of patients		
	All (n = 64)	Low ETV1 (n = 32)	High ETV1 (n = 32)
No mutation	4	3	1
KIT	58	27	31
**Exon 9**			
Insertion	2	2	0
**Exon 11**			
Insertion	9	2	7
Deletion	25	14	11
(Including codons 557–558)	(10)	(7)	(3)
Point mutation	21	7	14
**Exon 17**			
Point mutation	1	1	0
PDGFRA	2	2	0
**Exon 18**			
Point mutation	2	2	0

### ETV1 mRNA expression in GISTs

**Figure 1:**
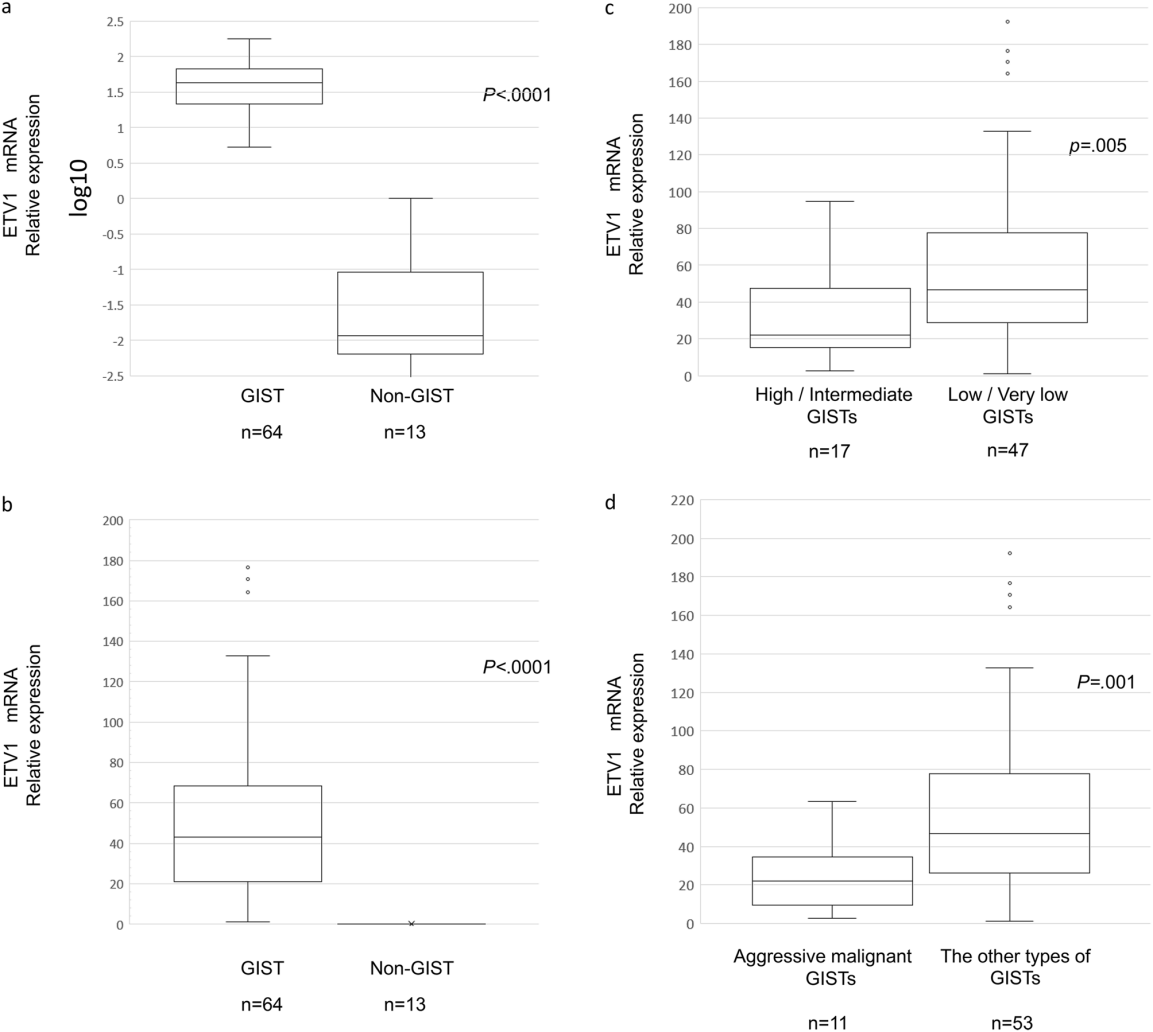
(**a**,**b**) Comparison of ETV1 mRNA expression in GISTs and non-GISTs. The data are shown on a semilogarithmic graph to base 10 (**a**) and on a linear graph (**b**). Patients with GISTs showed significantly higher relative ETV1 mRNA expression levels than those without GISTs (*P* < 0.0001). The comparison of two groups was performed using the Mann–Whitney U test. Error bars represent the standard deviation. (**c**) Comparison of ETV1 mRNA expression between high- or intermediate-risk GISTs and low- or very low-risk GISTs. ETV1 mRNA expression was higher in patients with very low- or low-risk GISTs than in those with intermediate- or high-risk GISTs (*P* = 0.005). The comparison of two groups was performed using the Mann–Whitney U test. Error bars represent the standard deviation. (**d**) Comparison of ETV1 mRNA expression between aggressive malignant GISTs and other types of GISTs. ETV1 mRNA expression was higher in patients with other types of GISTs than in those with aggressive malignant GISTs (*P* = 0.001). The comparison of two groups was performed using the Mann–Whitney U test. Error bars represent the standard deviation.

GISTs exhibited significantly higher relative ETV1 mRNA expression levels (median: 44.3, range 1.0–192.3) than non-GISTs (median: 0.008, range: 0.0–0.13) (*P* < 0.0001) (Fig. 1a,b). The minimum ETV1 expression level in the GISTs was higher than the maximum level in the non-GISTs.

To investigate whether ETV1 is an indicator of an aggressive malignant GIST, we examined the relationships between ETV1 mRNA expression and risk classification. ETV1 mRNA was relatively highly expressed in very low- and low-risk GISTs (n = 47) (median: 46.7, range 1.0–192.3) compared with intermediate- and high-risk GISTs (n = 17) (median: 21.9, range 2.6–94.8) (*P* = 0.005) (Fig. 1c). In addition, the aggressive malignant GISTs (n = 11) exhibited significantly lower ETV1 mRNA levels (median: 23.2, range 2.6–51.5) than the other types of GISTs (n = 53) (median: 47.7, range 1.0–95.7) (*P* = 0.001) (Fig. 1d). When the protein expression of ETV1 in the three groups was examined by Western blot, ETV1 expression was detected only in GISTs. One GIST in the low ETV1 group did not display protein expression, and the others expressed less protein than those in the high ETV1 group (Supplementary Fig. S1).

### Low ETV1 expression was associated with recurrence after surgery

Regarding postoperative recurrence, ETV1 mRNA expression was a significant factor in the univariate analysis (Table 3). On the other hand, the mutation type, such as the KIT exon 11 deletion, was not identified as a significant factor. The multivariate analysis showed that ETV1 expression (hazard ratio [HR] 8.1; 95% confidence interval [CI] 1.4–48.0, *P* = 0.022), tumor size (HR 4.8; 95% CI 1.1–21.3, *P* = 0.039), location (HR 11.7; 95% CI 1.5–88.0, *P* = 0.017) and mitotic count (HR 13.9; 95% CI 2.8–69.0, *P* < 0.001) were independent factors for recurrence after surgery. Regarding OS, only a mitotic count over 10/5 mm^2^ after surgery was selected as a significant factor.

**Table 3:** Univariate and multivariate analyses regarding postoperative outcomes (n = 64).

	Postoperative recurrence						Overall survival		
	Univariate analysis			Multivariate analysis			Univariate analysis		
	HR	95% CI	P-value	HR	95% CI	P-value	HR	95% CI	P-value
**Tumor size**									
< 50	1 (reference)	–	0.001	1 (reference)		0.039			0.353
> = 50	7.9	2.3–27.1		4.8	1.1–21.3				
**Mitotic count /5 mm**^**2**^									
< 10	1 (reference)	–	< 0.001	1 (reference)	–	< 0.001	1 (reference)		0.001
> = 10	14.8	4.5–49.7		13.9	2.8–69.0		23.4	3.8–142.0	
**Location**									
Stomach	1 (reference)	–	0.004	1 (reference)	–	0.017			0.054
Other	9.7	2.0–46.5		11.7	1.5–88.0				
**KIT exon 11 deletion**									
Present			0.269						0.073
Absent									
**Point mutation in KIT or PDGFRAPoint mutation in KIT or PDGFRA**									
Present			0.172						0.111
Absent									
**ETV1**									
High	1 (reference)	–	0.043	1 (reference)	–	0.022			0.183
Low	4.9	1.1–22.4		8.1	1.4–48.0				

We then examined the relationships between ETV1 mRNA expression and OS or RFS.

**Figure 2:**
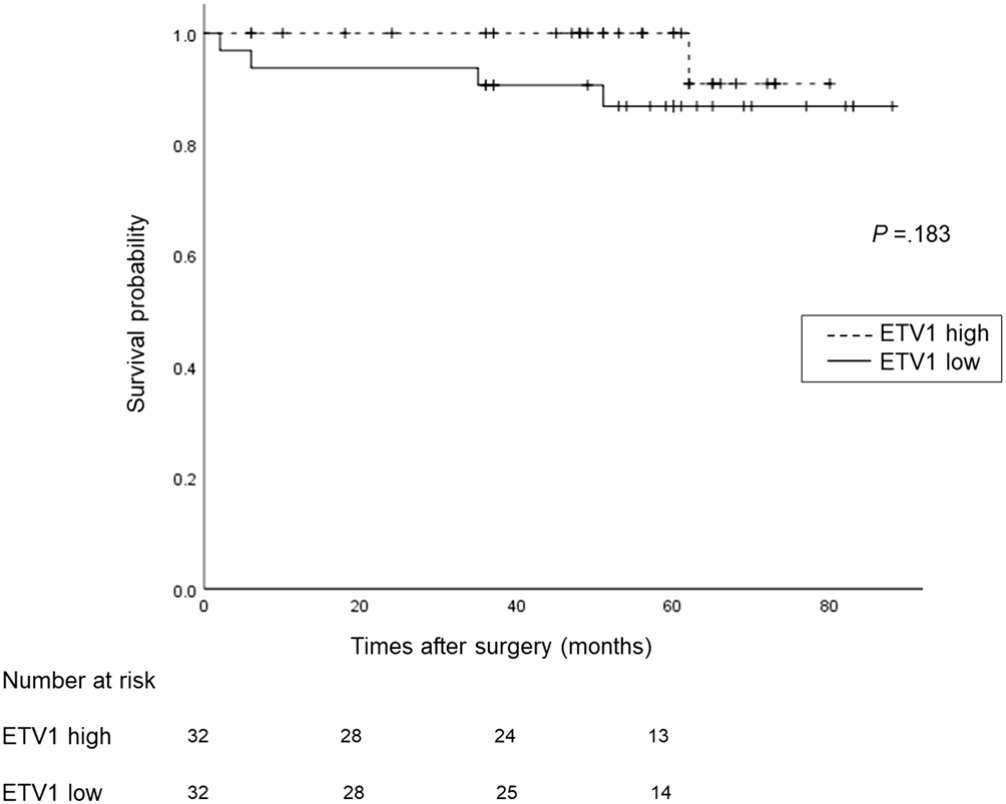
Kaplan–Meier curve of survival probability stratified by high or low ETV1 expression. The 5-year OS rate was 90.9 ± 8.7% in the high ETV1 expression group and 86.8 ± 6.2% in the low ETV1 expression group. There was no significant difference between the two groups (*P* = 0.270).

**Figure 3:**
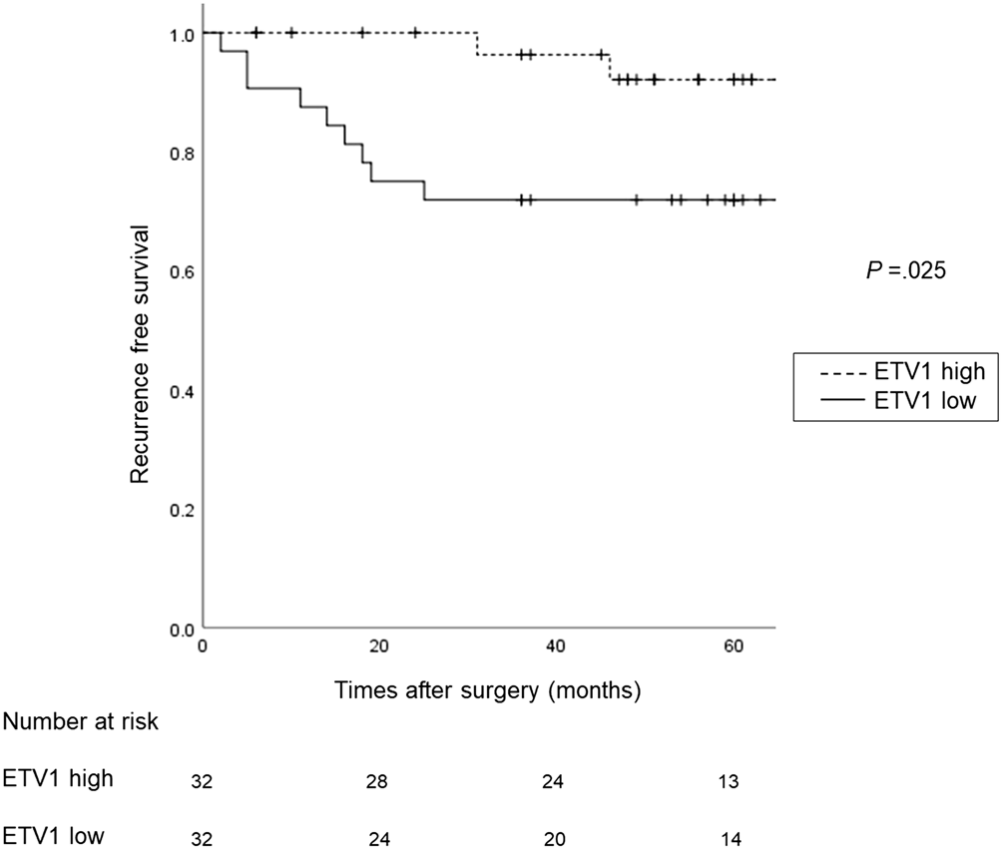
Kaplan–Meier curve of recurrence-free survival stratified by high or low ETV1 expression. Patients in the high ETV1 expression group displayed significantly prolonged RFS compared with patients in the low ETV1 expression group (*P* = 0.025). The 5-year RFS rate was 92.1 ± 5.4% in the high ETV1 expression group and 71.8 ± 7.9% in the low ETV1 expression group.

Sixty-four patients with GISTs were assigned to the low (n = 32) or high (n = 32) ETV1 expression level groups based on the median ETV1 mRNA expression level (cut-off: 44.3) in all GISTs. The clinical features and mutation status of each group are shown in Tables 1 and 2, respectively. As shown in Fig. 2, the 5-year OS rate was 90.9 ± 8.7% in the high ETV1 group and 86.8 ± 6.2% in the low ETV1 group. There was no significant difference between groups (*P* = 0.183). On the other hand, patients in the high ETV1 group displayed significantly prolonged RFS compared with patients in the low ETV1 group (*P* = 0.025) (Fig. 3). The 5-year RFS rate was 92.1 ± 5.4% in the high ETV1 group and 71.8 ± 7.9% in the low ETV1 group. The mean RFS time was 63 months (95% CI 52–74) in the low ETV1 group and 77 months (95% CI 72–81) in the high ETV1 group (HR 4.8; 95% CI 1.1–22.4; *P* = 0.043). Nine of the 32 (28%) patients in the low ETV1 group developed recurrent disease during the follow-up period, whereas only two patients (6.3%) in the high ETV1 group experienced recurrence.

## Discussion

Although numerous studies of GISTs have been conducted to discover the genetic and molecular mechanisms responsible for the disease, no critical biomarkers that can be used to predict malignancy have been identified. Here, we clarified the relationships between ETV1 expression and GISTs. First, we found that ETV1 expression was markedly upregulated at the mRNA level in GISTs. Second, ETV1 mRNA expression was significantly attenuated in aggressive malignant GISTs. Third, the multivariate analysis showed that ETV1 expression, in addition to conventional risk factors, such as tumor size, location and mitotic count, was an independent factor of recurrence. Moreover, the patients with GISTs that exhibited low ETV1 expression experienced shorter RFS than patients with GISTs that exhibited high ETV1 expression. Thus, ETV1 mRNA expression is negatively associated with malignancy in GISTs.

After it was first suggested that a relationship might exist between ETV1 expression and GISTs^15^, several researchers examined ETV1 expression in GISTs using immunohistochemical (IHC) staining and reported that it was present in approximately 50–60% of GISTs^16,17^, which disagreed with the findings of the initial study^15^. In the present study, real-time PCR analysis confirmed that ETV1 mRNA was expressed in all GISTs. On the other hand, non-GISTs (adenocarcinoma, schwannoma and leiomyoma) exhibited significantly lower ETV1 mRNA expression than GISTs. A similar result was reported in 2019: ETV1 expression was significantly higher in GISTs than in other sarcomas^20^. This discrepancy in positive frequency may depend on the detectability of the examination, such as IHC staining or RT-qPCR. Our finding that all GISTs express ETV1 mRNA universally supports that ETV1 is related to GIST formation. Furthermore, we determined that ETV1 mRNA expression was related to clinical outcomes in patients with GISTs, i.e., an inverse relationship was detected between ETV1 mRNA expression and RFS. This result implies that ETV1 expression is a useful molecular marker in GISTs.

After ETV1 was reported to contribute to GIST proliferation in cooperation with KIT in 2010, several studies on ETV1 expression in GISTs were published^17,21–23^. However, there is no consensus about ETV1 expression in GISTs. One of the reasons may be the evaluation method using IHC staining, which depends on the affinity of the antibody. On the other hand, there is only one report regarding mRNA, in which 95% of GISTs and 5% of non-GISTs were positive for ETV1 mRNA in situ hybridization^21^. However, our assessment of ETV1 mRNA expression using real-time PCR was different and showed that ETV1 was ubiquitously expressed at the mRNA level in GISTs, and low ETV1 expression was associated with poor RFS. However, there was no difference in OS between the low and high ETV1 expression groups. Eleven patients who experienced recurrence were treated with TKIs. Although two patients died within one month, five patients achieved 5-year survival. We presume that the development of molecular targeted therapies such as imatinib, sunitinib, and regorafenib might explain the marked discrepancy between OS and RFS in patients with GISTs.

The role of ETV1 in GISTs implied in the current study may be controversial. However, our finding applies to only ETV1 mRNA, which is completely different from the ETV1 protein. Moreover, protein expression does not always reflect mRNA expression. There are several reports that ETV1 at the protein level is stabilized by activated KIT signaling^15,24,25^. Therefore, a negative feedback system to regulate mRNA by the ETV1 protein might explain both our and previous results.

There are some limitations to this study. First, the sample size was small, although several data showed a statistically significant difference. Second, the cut-off value used in this study to distinguish between high and low expression was decided by the median. The value was not an absolute value and may have been altered by the study population, especially the rate of high-risk GISTs. Third, most GISTs in this study originated from the stomach. Our results may be applied only to gastric GISTs. Fourth, the protein level of ETV1 expression was not assessed simultaneously by Western blotting in all cases. Fifth, the molecular mechanism of attenuated ETV1 expression was not examined in this study. Further experimental and clinical studies are necessary to more clearly reveal the relationship between ETV1 expression and the aggressiveness of GISTs.

In conclusion, low ETV1 expression was associated with worse RFS and was revealed as an independent factor of recurrence in addition to conventional factors. ETV1 expression could be a predictor of aggressive malignant GISTs. An evaluation system based on ETV1 expression may help choose patients who need adjuvant chemotherapy.

## Supplementary information

Supplementary information

## Abbreviations

GIST: Gastrointestinal stromal tumor
ETV1: ETS translocation variant 1
PCR: Polymerase chain reaction
PDGFRA: Platelet-derived growth factor receptor A
NIH: National Institutes of Health
OS: Overall survival
RFS: Recurrence-free survival
IHC: Immunohistochemical
