# Supplementary material for: Low ETV1 mRNA expression is associated with recurrence in gastrointestinal stromal tumors
Source: Sci Rep. 2020 Sep 8;10:14767. doi: 10.1038/s41598-020-71719-y (PMC7478956; doi:10.1038/s41598-020-71719-y)

Title

Low ETV1 mRNA expression is associated with recurrence in gastrointestinal stromal tumors

Authors

Keiichi Sakamaki, MD [1], Kohei Funasaka, MD, PhD [1,2] *, Ryoji Miyahara, MD, PhD [1], Kazuhiro Furukawa, MD, PhD [1], Takeshi Yamamura, MD, PhD [1], Eizaburo Ohno, MD, PhD [1], Masanao Nakamura, MD, PhD [1], Hiroki Kawashima, MD, PhD [1], Yoshiki Hirooka, MD, PhD [1], Mitsuhiro Fujishiro, MD, PhD [1], Hidemi Goto, MD, PhD [1].

[1] Department of Gastroenterology and Hepatology, Nagoya University Graduate School of Medicine, 466-8550 Nagoya, Japan

[2] Department of Gastroenterology, Fujita Health University School of Medicine, 470-1192 Toyoake, Japan

* To whom correspondence should be addressed: Kohei Funasaka

Department of Gastroenterology, Fujita Health University School of Medicine

1-98 Kutsukake-cho, Toyoake, Aichi 470-1192, Japan

E-mail: k-funa@med.nagoya-u.ac.jp

Telephone: 81-562-93-9240; Fax: 81-562-93-8300

Supplementary Figure 1

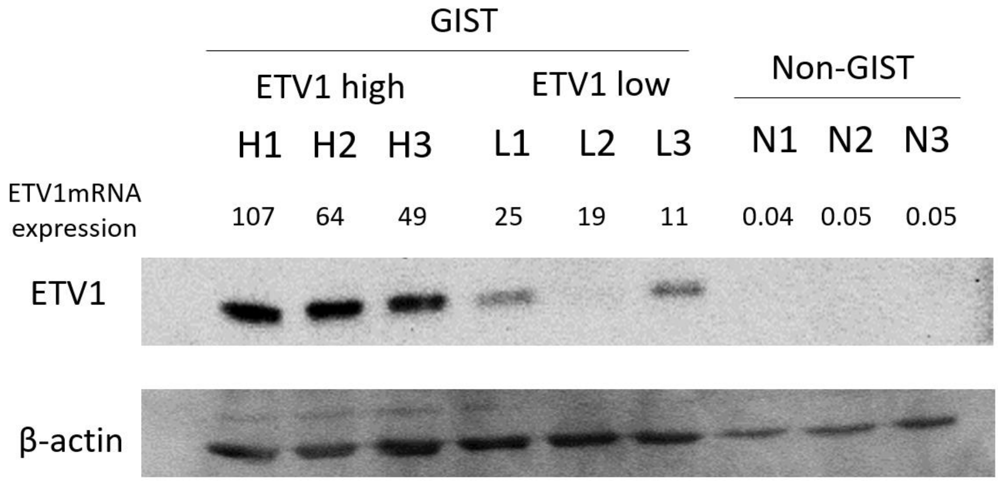

Supplementary Figure 1; The comparison of protein expression among three groups (GISTs with high ETV1 mRNA expression, those with low and non-GIST). The value which is described beneath each sample name shows the relative expression of ETV1 mRNA.

Supplementary Table1

Primer design for PCR

target cDNA	forward	reverse
KIT	AGCTCGGATCCCATCGCTA	CTGCTCAGACATCGTCGTGCAC
PDGFRA	ATCCGGCGTTCCTGGTCTTAG	CAGGAAGCTGTCTTCCACCAG

Primer design for sequence PCR

target		
KIT	exon8	CACCGAAGGAGGCACTTACA
	exon 9	GTGAATGGCATGCTCCAATGT
	exons 11 & 13	TCACTCCTTTGCTGATTGGT
	exon 17	ATCATGGAGGATGACGAGTTG
PDGFRA	exon 12	ATCTCACTTATTGTCCTGGTT
	exon 14	AGCCGGTCCCAACCTGTCATG

	exon 18	AGCTTCACCTATCAAGTTGCC

Supplementary Table1; Primer designs for PCR amplification and direct sequencing of KIT/ PDGFRA in clinical sample.

Supplementary Table2

Table S2.

Characteristics of patients died of GIST

No	gender	origin	tumor size (mm)	NIH-risk	mutation gene	mutation position	mutation type	ETV1 high/low grouping	Adjuvant TKI	RFS (months)	Recurrence	TKI after recurrence	OS (months)
1	M	stomach	80	high	KIT	exon11codon557-558	deletion	low	I	16	P	S	35
2	F	small intestine	300	high	none	-	-	low	-	1	P	I	2
3	F	stomach	200	high	KIT	exon11codon558-564	deletion	low	-	5	P	I	6
4	F	stomach	75	high	KIT	exon11codon557-558	deletion	high	-	31	L	I	62
5	F	stomach	195	high	KIT	exon11codon561-562	deletion	low	-	14	L	I, S, R	51

Abbreviation: RFS; recurrence-free survival, OS; overall survival, I; imatinib, S; sunitinib, R; regorafenib, P; peritoneal dissemination, L; liver metastasis

Supplementary Table2; Characteristics of five patients who died of GIST.

The original unprocessed images for western blot and the target panels were marked in red boxes.

ETV1

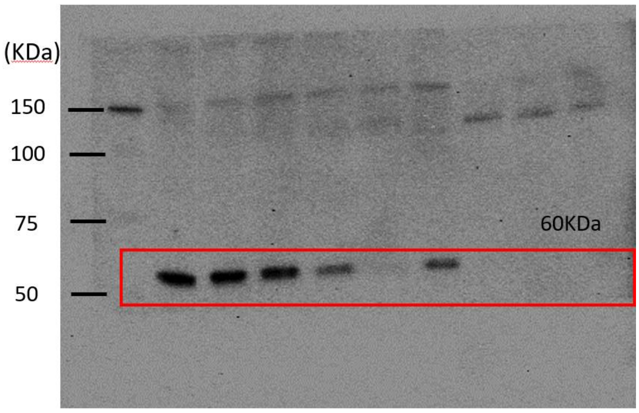

β-actin

42KDa

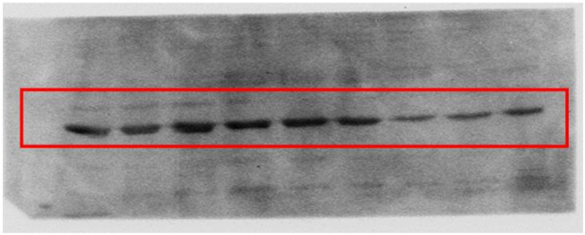

Supplement: Supplementary file 1 — Supplementary information [file 41598_2020_71719_MOESM1_ESM.pdf]
